# Screening Strategies to Improve Early Diagnosis in Endometrial Cancer

**DOI:** 10.3390/jcm13185445

**Published:** 2024-09-13

**Authors:** Silvia Cabrera, Irene de la Calle, Sonia Baulies, Antonio Gil-Moreno, Eva Colas

**Affiliations:** 1 Gynecologic Oncology Unit, Department of Gynecology and Obstetrics, Hospital de la Santa Creu i Sant Pau, 08025 Barcelona, Spain; 2 Fundación Santiago Dexeus Font, Gynecology, Obstetrics and Reproductive Medicine Department, Dexeus Mujer, 08028 Barcelona, Spain; sonbau@dexeus.com; 3 Research Group in Gynecology, Vall d’Hebron Institut de Recerca, 08035 Barcelona, Spain; irene.delacalle@vhir.org (I.d.l.C.); antonio.gil@vallhebron.cat (A.G.-M.); eva.colas@vhir.org (E.C.)

**Keywords:** endometrial cancer, diagnosis, screening, high-risk population

## Abstract

Endometrial cancer is the most common gynecological malignancy in high-income countries and the sixth most common cancer in women. Overall incidence has risen in the last few decades as a consequence of the increase in the prevalence of its risk factors, mainly obesity and the aging of the population, and although diagnoses have increased across all age groups, the incidence rates have doubled in women under the age of 40 years. The survival rates of endometrial cancer are highly dependent on its stage at diagnosis, bringing to the fore the importance of early diagnosis. The aim of a screening strategy in this type of tumor should be to detect the disease in the pre-invasive or early stage (before developing myometrial invasion), which would improve cure rates, reduce the morbidity associated with aggressive treatment and offer uterus-sparing management options for younger women. The ideal screening tool in this scenario would be a minimally invasive, inexpensive and easy-to-perform test or auto-test, which could be implemented in a routine gynecologic checkup of patients at-risk or in the general adult population. In this comprehensive review, we aim to define the populations at higher risk of developing endometrial cancer, to assess the performance of current diagnostic tools when used in a screening setting and to discuss the accuracy of new molecular screening strategies.

## 1. Introduction

Endometrial cancer (EC) is the most common gynecological malignancy in high-income countries and the sixth most common cancer in women [1]. Overall incidence has risen by 132% in the last 30 years, as a consequence of the increase in the prevalence of risk factors, mainly obesity and aging [2], and an significant rise in incidence is expected over the next few decades [3]. Although diagnoses have increased across all age groups, the incidence rates have doubled in women under the age of 40 years [4]. The survival rates of EC are highly dependent on its stage at diagnosis, falling from 95% for stage I to 14% for stage IV [3], bringing to the fore the importance of early diagnosis. In addition, the clinical profile of EC patients, frequently those elderly, obese or bearing associated morbidity, reveals the expected impact of less extensive therapies in their quality of life. 

A screening strategy in EC should be to detect the disease in the pre-invasive stage (atypical hyperplasia or intraepithelial atypical neoplasia) or initial stage (intramucosal stage). An early stage at diagnosis is expected to improve cure rates, reduce the morbidity related to aggressive treatment and offer uterus-sparing management options for younger women wishing to preserve their fertility [3,5]. The ideal screening tool in this scenario would be a minimally invasive, inexpensive and easy-to-perform test that could be implemented in a routine gynecologic checkup of patients at-risk [6]. Specifically, in EC, a screening tool should also be specific enough to rule out the presence of cancer in symptomatic patients, such as the numerous patients complaining about abnormal uterine bleeding (AUB). 

The aim of this review is to identify the populations at higher risk of having EC, who could become the target population of a screening strategy, as well as to assess the performance of current diagnostic tools when used in a screening setting and to review the accuracy of new screening strategies. 

## 2. Target Population for Screening Strategies

The lifetime risk of EC in the general female population is 2%, but this risk will be heavily modified by known risk factors [7]. Identifying high-risk individuals for targeted screening is a priority to implement screening strategies with good cost-effective results (Table 1) [8]. Around 85% of EC cases will appear in women older than 55 years old, being age the main risk factor for EC [5,9]. Nevertheless, the screening of the entire menopausal population would require a massive amount of funding; therefore, there is a need to identify subgroups of women at high-risk of suffering from EC in order for it to be cost-effective [10].

### 2.1. Genetic Risk

Lynch syndrome is an autosomal dominant inherited condition of defective DNA mismatch repair, which affects the MSH2, MLH1, MSH6 and PMS2 genes [23]. Women with Lynch syndrome have a 25–60% lifetime risk of EC and they develop it at younger ages than women with sporadic EC [5,11,12]. Current screening strategies for these women are annual transvaginal ultrasonography and endometrial biopsy after the age of 35 years; however, the evidence supporting this strategy is limited [24,25].

The EC risk estimates for Cowden syndrome (PTEN mutation) are inconsistent, although the lifetime risk is considered increased. EC was found in 14.1% and 7.6% of female PTEN mutation carriers in two large research series [26,27], and in 17% of clinically tested adult females in another cohort, with the greatest increase in women under the age of 50 [27]. An increased lifetime risk of EC in PTEN mutation carriers ranging from 19 to 28% at age 70 has been reported [13,14]. 

Evidence reporting data on endometrial cancer incidence in BRCA mutation carriers is confusing and sometimes conflicting. A systematic review and meta-analysis recently evaluated the EC risk in more than 13.800 BRCA1/2 mutation carriers. EC prevalence was 0.62% in BRCA1 mutation carriers and 0.47% in BRCA2 mutation carriers, with a relative risk of 1.18 (95% CI, 0.7–2.0). For serous EC, the prevalence was 0.2% and 0.08% among BRCA1 and BRCA2 mutation carriers, respectively, with a relative risk of 1.39 (95% CI, 0.5–3.7). However, although there were no significant differences in their meta-analysis, the authors report that, in most of the studies included in their meta-analysis, they observed a slightly increased risk of EC in BRCA mutation carriers, mainly for BRCA1 women, and individualized counseling was recommended [15]. 

For women who tested negative for hereditary known mutations, the concept of a polygenic risk score (PRS) based on genetic variants determined by genome-wide association studies would be of potential interest, since it can predict women who are at higher risk of developing EC [28]. A first-degree relative with EC entails a double risk of the disease even when a specific genetic variant is not detected [29], and a considerable part of this familial risk can be explained by common single nucleotide polymorphisms [30,31].

### 2.2. Obesity 

EC is the neoplasm that has the strongest link with obesity [32], with every additional 5 kg/m^2^ of body mass index (BMI) associated with a 60% increased risk of developing an EC (95% CI, 40–60%) [33]. The 2% estimated lifetime risk of EC in general population increases until 9–10% in obese patients [8], and 35% of all EC have been directly attributed to obesity [34]. A meta-analysis of seven prospective studies showed that, compared with normal-weighted women, the risk of EC is increased linearly by 1.5-fold among those who are overweight (BMI ≥ 25 and <30 kg/m^2^), by 2.5-fold among those with class 1 obesity (BMI ≥ 30 and <35 kg/m^2^), by 4.5-fold among those with class 2 obesity (BMI ≥ 35 and <40 kg/m^2^), and by 7.1-fold among those with class 3 obesity (BMI ≥ 40 kg/m^2^, lifetime risk 10–15%). The incidence of cancer in these patients is higher for endometrioid histology (16–23% per 2-unit increase in BMI) than for non-endometrioid tumors (12% per 2-unit increase in BMI) [7]. A positive association has also been described between central adiposity, as reflected by waist circumference (HR per 1-SD increase = 1.08, 95% CI: 1.00–1.17) and waist to hip ratio (HR per 1-SD increase = 1.13, 95% CI: 1.01–1.26) and EC risk after accounting for BMI [35]. 

### 2.3. Metabolic Syndromes

Insulin resistance, hyperinsulinemia, type 2 diabetes, and polycystic ovarian syndrome (PCOS) are known to promote endometrial proliferation by reducing the levels of sex hormone binding globulin and insulin-like growth factor 1 (IGF-1) binding proteins. This fact provokes an increased bioavailability of both estrogen and IGF-1, leading to an activation of the pro-oncogenic PI3K–AKT–mTOR signaling pathway, which entails endometrial proliferation. These mechanisms are activated independently of the presence of obesity in these patients [36]. 

Type 2 diabetes mellitus confers a 62% increased risk of EC after controlling for obesity in large epidemiologic studies [16]. Women with PCOS also show an increased risk of 9% when comparing with general population [17]. This risk remains higher after controlling for BMI (OR 2.79; 95% CI, 1.31–5.96) and, when selecting only women aged under 54 years, the EC risk in women with PCOS increased further (OR 4.05; 95% CI, 2.42–6.76) [18]. Taking into account the high prevalence of these two metabolic disorders in female population, they should be considered as important risk factors when selecting high-risk populations of EC patients.

### 2.4. Tamoxifen and Hormonal Therapies

The use of menopausal hormonal therapy has been associated with both an increase [37] and a decrease [7] in EC risk. Recent data report the safety of combined hormonal therapy when progestins are administered more than 10 days each month [3]. The use of menopausal hormonal therapy modifies the risk associated with BMI, with the risk being higher for women who have never used hormonal therapy (90% increased risk per 5-unit increase in BMI) than for those who have ever used hormonal therapy (18% per 5-unit increase in BMI) [7].

Breast cancer survivors receiving tamoxifen treatment also have an increased risk of developing EC. Tamoxifen is a selective estrogen receptor (ER) modulator, with an antagonistic effect on the α-ER in breast tissue and agonistic effects on the β-ER in the endometrium. It is associated with an increased incidence of endometrial abnormalities, including hyperplasia, atypia and malignancy. This increased incidence is dose and duration dependent, as was reported in a meta-analysis including four randomized clinical trials, which showed a two-fold increased risk with extended tamoxifen therapy compared with the standard 5 years of tamoxifen (RR 2.29, 95% CI 1.60 to 3.28; *p* < 0.001) [19]. 

### 2.5. Women with Abnormal Uterine Bleeding (AUB) 

**Figure 1:**
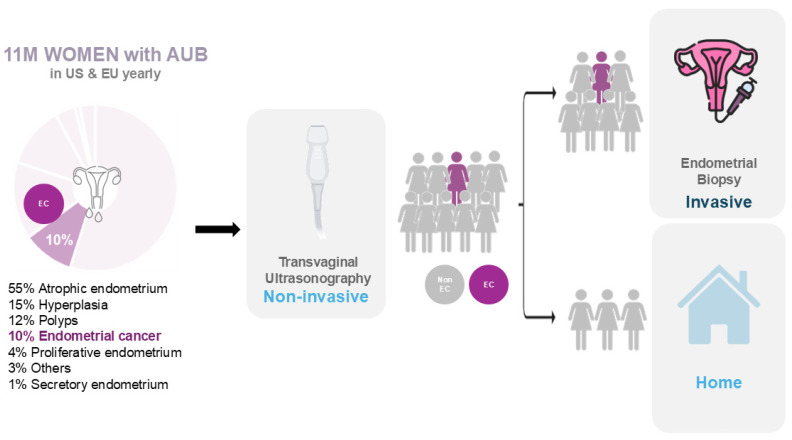
Diagnostic pathway of endometrial cancer of women with postmenopausal bleeding.

Although postmenopausal bleeding is considered an early symptom of EC [22], AUB in menopausal women is a very common complaint, accounting for a 5% of gynecologic consultations [22]. Only 8–11% of women with postmenopausal bleeding will finally receive a diagnostic of EC [20,21], and this incidence will also vary depending on age, being less than 1% in women younger than 50 years, rising to 3% in those aged 55 years and 24% in those older than 80 years [8]. The high incidence of EC in patients with postmenopausal bleeding leads to the fact that thousands of patients will require an endometrial test to rule out the presence of endometrial cancer every year (Figure 1). AUB is even more frequent in premenopausal women, where heavy, prolonged, or intermenstrual bleeding are extremely common complaints and caused by EC in just 0.3% of cases [22]. In this age range, the low incidence of EC entails frequent diagnostic delays [9,10]. In this scenario, the implementation of a screening test in women presenting with AUB that could identify patients with EC and safely rule out healthy patients will spare most of invasive endometrial tests and diagnostic delays.

## 3. Performance of Diagnostic Tools When Assessed in a Screening Setting

Some authors have assessed the use of diagnostic tools when used in a setting of EC screening. We review the performance of ultrasonography, cervico-vaginal and uro-vaginal cytologies to early detect EC (Table 2). 

### 3.1. Transvaginal Ultrasonography

Transvaginal ultrasonography provides a non-invasive assessment of endometrial thickness, which is used for triage of women who require further investigations. Analyzing the results of 48,230 women enrolled in the United Kingdom Collaborative Trial of Ovarian Cancer Screening (UKCTROC), a sensitivity of 80.5% (95% CI 72.7–86.8) and specificity of 86.2% (85.8–86.6) were reported using an endometrial thickness cut-off of 5.15 mm. When the analysis was restricted to asymptomatic, postmenopausal women with EC or atypical hyperplasia, a cutoff of 5 mm achieved a sensitivity of 77.1% (67.8–84.3) and specificity of 85.8% (85.7–85.9) [38]. 

Van der Bosch et al. assessed endometrial thickness in 2216 women (1373 premenopausal and 843 postmenopausal) presenting with different gynecological disorders. Women with EC had a mean endometrial thickness of 16.4 mm (95% CI 14.8–18.1 mm), compared with 4.1 mm (95% CI 3.5–4.7 mm) for those investigated but found not to have EC. Nevertheless, other benign disorders such as hyperplasia without atypia or endometrial polyp showed a median endometrium thickness of 12.1 mm and 10 mm, respectively [42]. In a meta-analysis of 44 studies including 17,339 women with AUB, a cut-off of ≥5 mm was considered the more appropriate threshold for further workup, due to its comparable sensitivity and negative predictive values (NPV) and increased specificity compared to less stringent cut-offs. The sensitivity of these thresholds did not vary based on age or use of hormone replacement therapy [43]. 

Recently, a retrospective study aiming to examine the false-negative probability using endometrial thickness thresholds as a triage for EC diagnosis among black individuals enrolled 1494 patients. Applying the <5 mm threshold, there was an 11.4% probability of missing an EC. At the <4 mm threshold, the probability was 9.5%, and at <3 mm, it was 3.8%. The authors concluded that the transvaginal ultrasonography triage is not reliable among black adults at risk for EC [44].

### 3.2. Cervico-Vaginal Cytology 

The sensitivity of cervico-vaginal cytology to diagnose EC was recently assessed in a systematic review and meta-analysis including 6599 women diagnosed with EC [45]. Abnormal results were observed in 45% of patients, being the incidence of abnormal tests higher in non-endometrioid histology (77% vs. 44%), advanced FIGO stage (63% stage III-IV vs. 41% stage I-II), and in tumors with nodal and cervical involvement. The authors concluded that the evaluation of cell abnormalities in cervico-vaginal cytology lacks the sensitivity to diagnose EC precociously, therefore dismissing this test as a screening tool [39].

### 3.3. Uro-Vaginal Cytology

Recently the results of a cross-sectional diagnostic accuracy study were reported, including 103 women with known gynecological cancer and 113 with unexplained postmenopausal bleeding who provided urine and/or vaginal samples to perform cytology [40]. The combination achieved a sensitivity of 91.7% (95% CI, 85.0–96.1%) and a specificity of 88.8% (95% CI, 81.2–94.1%) for gynecological cancer detection. In women with unexplained postmenopausal bleeding, cytology identified all four EC of the cohort and also diagnosed three other cancers (cervical, ovarian and bladder), with an 11.2% false positive rate [40]. The diagnostic accuracy of uro-vaginal cytology is currently being prospectively tested in DETECT study [46], a multicenter diagnostic accuracy study of women with postmenopausal bleeding who require further studies to rule out or confirm EC. 

### 3.4. Endometrial Sampling

Although endometrial biopsy is the current standard of care for endometrial cancer diagnosis, it shows several disadvantages for its use in a screening setting. The main limitation across all outpatient endometrial sampling techniques, including pipelle biopsy, outpatient hysteroscopy or others, is patient acceptability due to pain and discomfort. Moreover, the difficulty in accessing the endometrial cavity due to cervical stenosis and atrophy entails an impossibility of sampling in 10% of cases. Finally, it is calculated that only 65% of the endometrial cavity is sampled with these outpatient techniques, carrying a concordance between an endometrial biopsy and a hysterectomy specimen of 60–70% [3,41].

## 4. New Biomarkers for Endometrial Cancer Screening 

The use of liquid biopsies as a source of biomarkers has been explored in EC. Multiple approaches have been employed in the search for screening and diagnostic biomarkers, such as proteomics, metabolomics and genomic sequencing [47], and different studies have deepened into the potential of blood, urine, uterine fluid or cervico-vaginal fluid as a source of biomarkers (Table 3). Some of these samples might enable self-sampling strategies; therefore, becoming promising screening tools [39,46]. 

### 4.1. Blood 

The main advantage of blood samples is their wide availability in routine clinical assistance. On the contrary, the main handicap is the low concentrations of biomarkers found in blood, especially in an early-stage disease. The most assessed protein biomarkers for diagnostic accuracy of EC are serum CA125 and Human Epididymis protein 4 (HE4), which have been reviewed by multiple studies and meta-analyses. Their performance is poor in terms of sensitivity and specificity, making them unsuitable for screening use [47]. A pilot study showed that using a serum HE4 cut-off value of 69.7 pmol/L to predict malignancy achieves a sensitivity of 86.7% with a specificity of 100% [49]. 

Regarding genomic biomarkers, the most described are related to the presence of tumor circulating material, as circulating tumor cells (CTCs), cell free DNA (cfDNA), cell-fee RNA (microRNA and long non-coding RNA) and exosomes [50]. The main limitation for their use is their low quantity present in circulation, despite the substantial improvement in highly sensitive techniques [59]. Gao et al. have recently assessed the use of circulating microRNA (miRNA) as an early diagnostic tool in an exhaustive review of published evidence, finding a sensitivity of 0.84 (95% CI: 0.79–0.88), a specificity of 0.87 (95% CI: 0.79–0.91) and an area under the ROC curve (AUC) of 0.91 (95% CI: 0.89–0.94), indicating that circulating miRNA had high diagnostic accuracy and is becoming a promising novel non-invasive biomarker for EC diagnosis [48].

Troisi et al. recently reported their results in the validation of a metabolomics signature as a screening tool in a large population of symptomatic women [51]. They performed the first validation on a cohort of 563 individuals and a second, independent validation with an enrollment of 871 women. Through a machine learning algorithm, they developed a screening test that showed an error rate of less than 5% in identifying EC. The authors concluded that metabolomics is a non-invasive, inexpensive, patient-friendly, and high-throughput technology that can simultaneously measure hundreds of different metabolites in a small volume of fluid or tissue, therefore becoming a useful screening tool for the early screening of EC. 

Paraskevaidi et al. published their results using blood-based spectroscopy and machine learning algorithms to differentiate healthy individuals from women with EC and atypical hyperplasia [60]. Spectroscopic techniques do not detect single biomarkers because the differential peaks may be formed by multiple biological substances. They analyzed blood plasma samples in a cross-sectional diagnostic accuracy study of women with EC (n = 342), atypical hyperplasia (n = 68) and healthy controls (n = 242). They reported 87% sensitivity and 78% specificity to detect EC, increasing to sensitivities of 91–100% and specificities of 81–88% when referring to endometrioid EC and atypical hyperplasia. The authors concluded that this strategy could enable the early detection of EC in symptomatic women and provide the basis of a screening tool in high-risk groups.

### 4.2. Urine 

Urine samples have the advantage of being non-invasive and easy to obtain, allowing for self-sampling. Urine CA125 and HE4 levels were assessed as protein biomarkers for early diagnostics in 153 symptomatic women through automated chemiluminescent enzyme immunoassays. They were found to be significantly higher in women with EC compared to controls, showing an AUC of 0.89 (95% CI 0.81, 0.98) and 0.69 (95% CI 0.55, 0.83), respectively. The authors developed a diagnostic model combining urine CA125 and a measurement of endometrial thickness by transvaginal ultrasound, achieving an AUC of 0.96 (95% CI 0.91, 1.00) for predicting EC [53]. 

Regarding genomic biomarkers, the performance of DNA methylation to detect EC in urine was assessed by Wever, along with cervicovaginal self-samples and clinician-taken cervical samples. Optimal three-marker combinations demonstrated excellent diagnostic performances with AUC values of 0.95 (95% CI: 0.92–0.98) in urine samples. This excellent performance was maintained for the stage I disease, showing that this approach has great potential to screen patient populations at risk for EC [52].

### 4.3. Uterine Fluid 

Uterine fluid has been demonstrated to be an interesting source of biomarkers, as it is in direct contact with the endometrial tumor and can be obtained easily by uterine aspiration. The main limitation to its use in a screening setting is that it is an invasive sample, and endocervical permeability is needed. However, taking into account that a uterine fluid sample does not need to contain tissue to be analyzed, it is expected that the failure rate of this specimen in the screening setting will be lower than that reported in diagnostics of EC [61]. 

Two genomic tests have been described for the early detection of EC. PapSEEK is a multiplex PCR-based test to detect genetic alterations—mutations in 18 genes as well as aneuploidy—in cervical or endometrial samples. Ninety-three percent (95% CI, 87–97%) of the endometrial samples from EC patients contained genetic alterations detected by PapSEEK, including 90% of patients with early-stage disease. No patient without cancer tested positive with PapSEEK in this study [54]. The kit Gynec-Dx^®^ consists of a five gene qRT-PCR assay performed in uterine samples. It was tested prospectively in a multicenter study including 514 women older than 45 years with AUB [55]. The combination of the histological and molecular analyses of the samples significantly increased the sensitivity (91%), specificity (97%) and NPV (99%) of EC diagnosis when compared to the results of histological analysis alone, and equaled the results obtained by hysteroscopy, considered as the gold standard for diagnostics in this setting.

With the aim of identifying proteomic biomarker signatures in uterine fluids, the levels of 52 proteins were measured in 116 women (69 EC patients, 47 controls). Twenty-eight proteins were found significantly higher in patients with EC, with a two-protein combination panel exhibiting a 94% sensitivity and 87% specificity for detecting EC cases, and a three-protein combination panel achieving a 95% sensitivity and 96% specificity for the discrimination of histologic subtypes [57]. These results need to be further validated. 

### 4.4. Cervico-Vaginal Fluid 

Cervical and vaginal samples seem to be an adequate source of EC biomarkers for screening. Their wide acceptance and good tolerance along with the options of self-sampling make them one of the best approaches when considering screening. 

Genomic biomarkers have shown the most promising results in cervico-vaginal samples. DNA methylation signature in vaginal fluid has been evaluated as an interesting tool to identify women with EC. The Women’s cancer risk IDentification for Endometrial Cancer (WID-qEC) is a three-marker test that evaluates DNA methylation in cervico-vaginal samples from symptomatic patients [56]. The WID-qEC identified 90.9% (95% CI, 70.8 to 98.9) of EC cases in samples, outperforming ultrasound in the detection of EC, predating diagnosis up to one year, and showing similar performance irrespective of the collection device and fluid, the sample collector (clinician or patient), or the precise sampling site [62]. The genetic test PapSEEK was also tested on cervical samples of 382 women with EC and 714 women without cancer. Eighty-one percent (95% CI, 77 to 85%) of the Pap brush samples from women with EC were PapSEEK-positive, including 78% of patients with early-stage disease. Only 1.4% of healthy controls tested positive in this study, showing a low false-negative rate [54]. 

Cervical fluids have been described as a rich source of protein biomarkers as well, and targeted proteomics identified SERPINH1, VIM, TAGLN, PPIA, CSE1L, and CTNNB1 as potential protein biomarkers to discriminate between EC and symptomatic controls in cervical fluids (AUC > 0.8) [58].

## 5. Conclusions

Endometrial cancer is the most frequent pelvic gynecological cancer in developed countries and shows a tendency of increasing incidence, mainly due to population aging and lifestyle changes. Patients diagnosed at an early stage show excellent outcomes, and current treatments are very effective in controlling the local disease with acceptable morbidity. On this premise, this malignancy is considered a good candidate for screening. However, in the case of EC, the possibility of detecting an early-stage disease in premenopausal, young women offers a window of opportunity to those who wish to preserve their fertility, becoming another important reason to investigate effective screening tools. 

Considering clinical risk factors, we identify some target high-risk groups that could widely benefit from EC screening strategies, listed as follows: (a) women with a known genetic syndrome, (b) women with class 2 or 3 obesity (BMI ≥ 35 kg/m^2^), (c) women of any age with abnormal uterine bleeding (AUB) and (d) pre-menopausal women with PCO and/or type 2 diabetes. None of the current diagnostic tests perform accurately when used in a screening setting; therefore, the discovery of new biomarkers seems to be the future for EC screening. Cervico-vaginal fluid seems the best sample to be investigated, as obtaining it is well-tolerated and widely accepted by patients in their routine gynecological check-up visits, and it allows for self-sampling for women who are willing to do so. Regarding the best biomarker combination, genomic and proteomic biomarkers showed promising results in previous studies performed in screening setting. 

Further investigation and funding should be promoted to discover the most accurate, inexpensive and easy to implement screening tool for endometrial cancer.

## Figures and Tables

**Table 1:** Population at-risk and reported lifetime incidence of endometrial cancer.

Target Population	Lifetime Incidence of Endometrial Cancer
General population [7]	2%
Lynch Syndrome [5,11,12]	25–60%
Cowden Syndrome [13,14]	19–28%
BRCA 1–2 [15]	2%
Overweight (BMI ≥ 25 and <30 kg/m^2^) [7]	3%
Class 1 obesity (BMI ≥ 30 and <35 kg/m^2^) [7]	5%
Class 2 obesity (BMI ≥ 35 and <40 kg/m^2^) [7]	9%
Class 3 obesity (BMI ≥ 40 kg/m^2^) [7]	10–15%
Type 2 DM [16]	3.5%
Premenopausal PCOS [17,18]	4%
Tamoxifen > 5 years [19]	4.5%
Postmenopausal AUB [20,21]	8–11%
Premenopausal AUB [22]	0.3%

**Table 2:** Diagnostic tools’ performance in a screening setting.

Screening Tool	Methodology	Accuracy
**Transvaginal Ultrasonography**	Measurement of transversal endometrial thickness	In asymptomatic, postmenopausal women, a cutoff of 5 mm achieved a sensitivity of 77.1% and specificity of 85.8% [38].
**Cervico-vaginal cytology**	Cytological assessment of cervico-vaginal samples	Sensitivity of 45% for EC [39].
**Uro-vaginal cytology**	Cytological assessment of self-collected vaginal and urine samples	In postmenopausal population with AUB, it has a sensitivity of 91.7% and a specificity of 88.8% for gynecological cancer detection [40].
**Endometrial sampling**	Histological assessment of endometrial biopsies obtained by pipelle or outpatient hysteroscopy	A total of 10% of failure to obtain; the concordance with hysterectomy specimen is 60–70% [3,41].

**Table 3:** New biomarkers and their performance in screening setting.

Sample	New Biomarkers	
Blood	Genomic biomarkers	Circulating microRNA (miRNA): sensitivity, 0.84; specificity, 0.87 and AUC 0.91 [48]
	Proteomic biomarkers	Human Epididymis protein 4 (HE4) > 69.7 pmol/L predicts malignancy with 86.7% sensitivity and 100% specificity [49] Spectroscopy and machine learning algorithms show 87% sensitivity and 78% specificity to detect endometrial cancer [50]
	Metabolomic biomarkers	A twelve metabolites signature showed an error rate of less than 5% in identifying EC [51]
Urine	Genomic biomarkers	DNA methylation: A 3-marker combination showed AUC 0.95 [52]
	Proteomic biomarkers	Urine CA125 and HE4 levels were discovered to be significantly elevated in women with EC compared to controls (*p* < 0.001 and *p* = 0.01, respectively), with AUC of 0.89 (0.81, 0.98) and 0.69 (0.55, 0.83), respectively [53]
Uterine fluids	Genomic biomarkers	PapSEEK is a multiplex PCR-based test to detect genetic alterations, with 93% sensitivity and 100% specificity [54]
		Gynec-Dx^®^ consists of a five-gene qRT-PCR assay that, combined with histological analyses, showed sensitivity 91%, specificity 97% and NPV 99% [55]
		WID-qEC is a 3-marker test that identifies 90.9% of EC patients [56]
	Proteomic biomarkers	Two-protein combination panel exhibits 94% sensitivity and 87% specificity [57]
Cervico-vaginal fluids	Genomic biomarkers	PapSEEK detected 81% of women with endometrial cancers, and only 1.4% of healthy controls tested positive [54]
	Proteomic biomarkers	Six proteins identified as potential biomarkers in cervical fluids (AUC > 0.8) [58]
